# A Field Evaluation of the Hardy TB MODS Kit™ for the Rapid Phenotypic Diagnosis of Tuberculosis and Multi-Drug Resistant Tuberculosis

**DOI:** 10.1371/journal.pone.0107258

**Published:** 2014-09-16

**Authors:** Laura Martin, Jorge Coronel, Dunia Faulx, Melissa Valdez, Mutsumi Metzler, Chris Crudder, Edith Castillo, Luz Caviedes, Louis Grandjean, Mitzi Rodriguez, Jon S. Friedland, Robert H. Gilman, David A. J. Moore

**Affiliations:** 1 Laboratorio de Investigación en Enfermedades Infecciosas, Universidad Peruana Cayetano Heredia, Lima, Peru; 2 Wellcome Centre for Clinical Tropical Medicine, Imperial College of Science, Technology and Medicine, London, United Kingdom; 3 PATH, Seattle, Washington, United States of America; 4 Laboratorio de Salud Pública de la Dirección Regional de Salud del Callao, Lima, Peru; 5 Johns Hopkins Bloomberg School of Public Health, Baltimore, Maryland, United States of America; 6 TB Centre and Department of Clinical Research, London School of Hygiene & Tropical Medicine, London, United Kingdom; The Catholic University of the Sacred Heart, Rome, Italy

## Abstract

**Background:**

Even though the WHO-endorsed, non-commercial MODS assay offers rapid, reliable TB liquid culture and phenotypic drug susceptibility testing (DST) at lower cost than any other diagnostic, uptake has been patchy. In part this reflects misperceptions about in-house assay quality assurance, but user convenience of one-stop procurement is also important. A commercial MODS kit was developed by Hardy Diagnostics (Santa Maria, CA, USA) with PATH (Seattle, WA, USA) to facilitate procurement, simplify procedures through readymade media, and enhance safety with a sealing silicone plate lid. Here we report the results from a large-scale field evaluation of the MODS kit in a government service laboratory.

**Methods & Findings:**

2446 sputum samples were cultured in parallel in Lowenstein-Jensen (LJ), conventional MODS and in the MODS kit. MODS kit DST was compared with conventional MODS (direct) DST and proportion method (indirect) DST. 778 samples (31.8%) were *Mycobacterium tuberculosis* culture-positive. Compared to conventional MODS the sensitivity, specificity, positive, and negative predictive values (95% confidence intervals) of the MODS Kit were 99.3% (98.3–99.8%), 98.3% (97.5–98.8%), 95.8% (94.0–97.1%), and 99.7% (99.3–99.9%). Median (interquartile ranges) time to culture-positivity (and rifampicin and isoniazid DST) was 10 (9–13) days for conventional MODS and 8.5 (7–11) for MODS Kit (p<0.01). Direct rifampicin and isoniazid DST in MODS kit was almost universally concordant with conventional MODS (97.9% agreement, 665/679 evaluable samples) and reference indirect DST (97.9% agreement, 687/702 evaluable samples).

**Conclusions:**

MODS kit delivers performance indistinguishable from conventional MODS and offers a convenient, affordable alternative with enhanced safety from the sealing silicone lid. The availability in the marketplace of this platform, which conforms to European standards (CE-marked), readily repurposed for second-line DST in the near future, provides a fresh opportunity for improving equity of access to TB diagnosis and first and second-line DST in settings where the need is greatest.

## Introduction

Global control of tuberculosis (TB) and multidrug resistant tuberculosis (MDRTB) is hindered by a lack of access to affordable and reliable diagnostic tests in the countries with the greatest need. As a result, avoidable transmission from undiagnosed individuals with active TB, MDR or otherwise, contributes significantly to the estimated 1.3 million people dying each year from this curable disease [1].

The microscopic-observation drug-susceptibility (MODS) assay, developed in Peru [2]–[4] is a highly sensitive and specific phenotypic diagnostic test for TB and MDRTB. This low-cost, non-commercial assay is well suited to resource-constrained settings and was endorsed by the WHO in 2010 [5]. Consumables for the MODS assay are available from well-known laboratory suppliers, and all standard operating procedures to perform the test are freely available in publications and on the internet [6]. However, the procurement by laboratory staff of reagents and consumables from multiple providers can be challenging, and settings with a high burden of TB tend to also be those more commonly afflicted by limited human and economic resources and unreliable supply chains due to inadequate infrastructure, weak administrative procedures, conflict and/or natural disaster [7]. In these circumstances mechanisms to simplify procurement, such as the availability of an “off the shelf” kit that requires interaction with only a single supplier, have appeal.

With this in mind Hardy Diagnostics [8] and the international non profit organization PATH [9], with technical input from the MODS team at Universidad Peruana Cayetano Heredia (UPCH) in Peru, have developed a kit version of the conventional MODS assay. The Hardy TB MODS Kit™ (referred to from here onwards as the “MODS Kit”) contains reconstituted enriched culture broth, antibiotic tablets that dissolve in reagent vials, and colour-coded step-by-step instructions to simplify and shorten the assay preparation process. The kit negates the requirement for separate procurement of the various components of the MODS assay and assures reagent quality control. Much of the time-consuming reagent and media preparation work is bypassed by the provision of ready-to-go materials in appropriate quantities.

Conventionally mycobacterial work, which entails manipulation of culture suspensions (such as for indirect drug susceptibility testing), requires biosafety level (BSL) category 3 laboratory facilities; however, conventional MODS, which exploits direct DST and therefore entails no routine manipulation of positive culture, can be performed under BSL2 conditions [10]. The MODS Kit incorporates a novel, specially designed, firm fitting transparent silicone lid that further alleviates previous biosafety and cross-contamination concerns related to the safe handling of liquid culture. The logistical and biosafety advantages of the kit over the conventional, non-commercial version of MODS are self-evident.

The purpose of this study was to determine whether the analytical performance of the MODS Kit was comparable to the high sensitivity and specificity that was widely reported using conventional MODS for detection of TB and MDRTB direct from sputum. Encouraged by pilot data from the research laboratory at Universidad Peruana Cayetano Heredia, we performed a head-to-head evaluation of the kit with conventional MODS in a government regional TB reference laboratory (DIRESA Callao) in metropolitan Lima, Peru.

## Methods

### Study Participants and Setting

The study was conducted in Lima, Peru, from July to December 2012 in the Callao regional TB reference laboratory. The study utilized sputum samples from patients with suspected TB in the catchment area of DIRESA Callao that were sent to the laboratory for routine TB culture and drug susceptibility testing (DST). Non-pulmonary samples or any sample containing less than 2 mL of sputum were excluded. No patient information was collected for the study.

### Laboratory Methods

#### Detection of M. tuberculosis

Sputum samples were decontaminated according to the standard N-acetyl-L-cysteine-sodium hydroxide (NALC-NaOH) method [11]. In a 50 ml conical tube 2–5 ml of collected sputum sample was homogenized with an equal volume of decontaminating solution (NaOH 4% mixed 1∶1 vol/vol with sodium citrate 2.9% then N-acetyl-L cysteine 0.5% for liquefaction) and left to stand for 15 minutes. The decontaminated sample was then mixed with 30 ml of phosphate buffer saline (PBS) and centrifuged at 3000 *g* for 15 minutes. The supernatant was discarded and a smear was prepared with 100 µl of the concentrated pellet for Ziehl-Neelsen staining and evaluated by microscopy. The remaining pellet was re-suspended with 1.5 ml of PBS and divided into equal thirds in vials (each containing 500 µl) for parallel Löwenstein-Jensen (LJ), conventional MODS and MODS Kit cultures. For each culture inoculum a back-up aliquot was stored at 4°C in case reprocessing was required due to culture contamination or an indeterminate culture reading. The contents of the first vial were inoculated onto an LJ slant (200 µl for the LJ slant and 300 µl for backup aliquot), incubated at 37°C and examined twice weekly from day 7 through day 60 for the presence of characteristic mycobacterial growth [12].

The second aliquot was used for the conventional MODS assay [2]–[4], [6]. Briefly, one vial containing 500 µl of sample pellet was re-suspended with 2 ml of Middlebrook 7H9 broth (BD), oleic acid, albumin, dextrose, and catalase (OADC) (BD), and polymyxin, amphotericin B, nalidixic acid, trimethoprim, and azlocillin (PANTA, BD), taken from one pre-prepared tube containing 5.1 ml (as per MODS SOP, www.modsperu.org). Then 1 ml of the re-suspended pellet was removed and stored as a backup. The remaining 1.5 ml was added to the remaining 3.1 ml of 7H9-OADC-PANTA and 900 µl of this final sample suspension was inoculated into each of four wells in a 24-well tissue-culture plate (BD) as per MODS SOP [6]. For each sample, four wells were used: in two drug free (diagnostic) wells no drug was added and each of the remaining two wells contained critical concentrations of either 1 µg/ml rifampicin or 0.4 µg/ml isoniazid, as per conventional MODS SOP. To minimize cross-contamination and occupational exposure, plates were permanently sealed inside plastic Ziploc bags after inoculation, placed in an incubator (non CO_2_ enriched) at 37°C, and were subsequently examined within the bag. The cultures were examined under an inverted light microscope at total magnification of 40X and 100X three times per week, on alternate days from the fifth day of incubation until day 21. Positive cultures were identified by cord formation, characteristic of *M. tuberculosis* growth, in liquid medium in drug-free control wells, as described previously [2]–[4], [6]. When such growth was observed in both the drug-free wells, the isoniazid and rifampicin containing wells were examined on the same day; the presence of growth in the presence of the critical concentration of drug was interpreted as resistance to that agent and the absence of growth as susceptibility, as previously described and validated [4].

The third aliquot of the reconstituted sputum sample was processed using the MODS Kit protocol. The 24 well kit plate was assembled with reagents provided within the MODS kit according to the colour coded instructions. Reagents provided are as follows: vials of Middlebrook 7H9 broth + OADC, a NAPTA™ tablet (containing an antimicrobial cocktail to minimise culture contamination) to be added to the broth (after reconstitution with provided vial of sterile water) and isoniazid and rifampicin tablets, reconstituted in 7H9-OADC vials. Inoculation of samples took place by the same method described above for the conventional MODS assay. Ziplock bags were not required because of the sealed silicone lid fitted to the plate. The reading frequency of plates and TB and DST diagnostic criteria were identical to those used for the conventional MODS assay. Technicians reading plates were blinded to the results of other tests and entered data into a database that was only linked with a corresponding database containing reference test results at the end of the study (database and database key available as Files S1 and S2 respectively). On each day that samples were processed by MODS Kit and conventional MODS, internal control positive strains (drug sensitive H37RV and wild type local clinical strains with well characterised isoniazid and rifampicin resistance) were employed and negative wells (containing enriched broth but no samples) were also set up and similarly read by both MODS methods.

If a sample was designated contaminated by any culture method, the back-up sample aliquot was decontaminated a second time by the same method and inoculated into culture once more. In the case of indeterminate culture results by either MODS method (conventional or kit), the sample was reprocessed using the same medium that gave the initial indeterminate result. The final result took into account these reprocessed samples. For positive TB results from any test, an aliquot of each isolate was stored at −70°C in 7H9-OADC glycerol to aid with later discrepant analysis.

For both MODS assay methods, the possible final outcomes were positive (≥2 colony-forming-units [CFU]/well in both drug-free wells), indeterminate (1 CFU/well in either drug-free well), negative (0 CFU/well), or contaminated. LJ culture was primarily undertaken to provide isolates for proportion method testing, though culture yields were compared with the MODS assays; in contrast to the stringent MODS criteria above, for LJ even a single colony was regarded as a positive diagnosis (thus there was no option to designate an indeterminate outcome); the remainder were classified either as negative or contaminated.

### Definition of drug susceptibility to rifampicin and/or isoniazid

Direct DST was performed in the conventional MODS assay, as previously described [2]–[4], [6]. Growth in drug-free control wells but not in drug-containing wells indicated susceptibility, and positive growth in the drug containing wells indicated resistance. In addition to the primary comparison between conventional MODS and the MODS Kit, the MODS Kit DST was further compared with indirect drug-susceptibility testing performed by an external laboratory using the proportion method [12]. This method uses isolates from the corresponding parallel LJ culture to compare the yield when strain is inoculated on both drug free and drug containing media. If the proportion of colonies cultured on drug containing media is more than 1% of that cultured on drug free media the strain is identified as resistant. The reference standard for comparison of the MODS Kit DST to indirect DST was the proportion method result; however, in the event of MODS Kit/proportion method discordance, a third arbiter test, the Genotype MTBDRplus (Hain) molecular line probe assay test, was used (again, by an external laboratory) for final discrepant analysis and this result was taken as the reference [13].

### Statistical Method

Data was analysed using Stata 11 (College Station, TX: StataCorp LP 2009). Sensitivity (the proportion of samples which tested positive by Mods Kit that tested positive by the gold standard test), specificity (the proportion of samples which tested negative by Mods Kit that were negative by the gold standard test), positive predictive value (the proportion of true positives in all samples that tested positive by MODS kit) and negative predictive value (the proportion of true negatives in all samples that tested negative by MODS kit) for both TB detection and MDRTB detection were calculated from the totals of true and false positive and negative results identified by the MODS Kit using conventional MODS as the reference assay. The TB detection results of the kit were also compared to both conventional MODS and LJ culture results in a Venn diagram. Median time to culture-positivity by method was compared using the Wilcoxon Rank Sum test.

A contamination rate for each method was calculated from the proportion of inoculated samples showing a contaminated culture, following initial inoculation and the final result after reprocessing the sample. The proportion of sample contamination for each method was compared using the Z-test for two proportions.

For each method, the rate of indeterminate samples was determined by calculating the proportion of plated samples with an indeterminate result, for both initial inoculation and the final designation obtained after re-inoculation of the sample. The proportion of indeterminate samples for each method was compared by the Z-test for two proportions.

A P-value of <0.05 was used to indicate statistical significance. The concordance of susceptibility results, with reference results defined as outlined above, was determined with the use of the sensitivity, specificity, and positive and negative predictive values for the detection of resistance with 95% confidence intervals, as well as with kappa values (A co-efficient that quantifies observed agreement between two tests, incorporating possible agreement by chance).

### Ethical Approval

The study was approved by the institutional review boards of Universidad Peruana Cayetano Heredia and DIRESA Callao.

## Results

### Sample Characteristics

**Figure 1:**
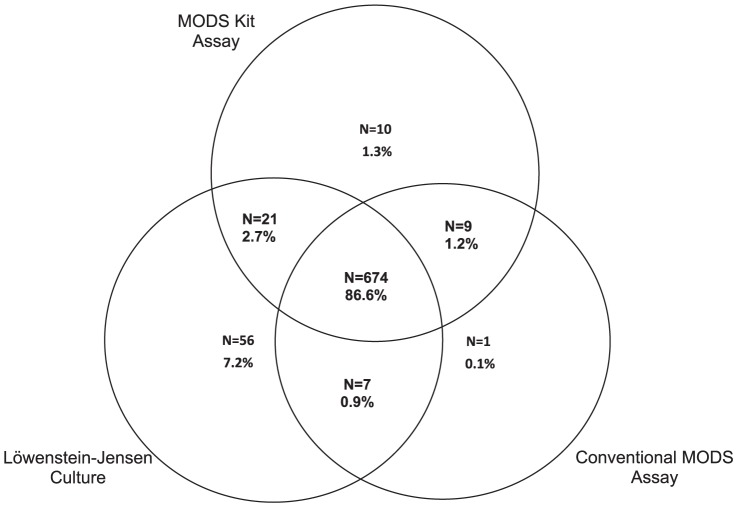
Proportion of samples positive by each culture method. Total samples: N = 2446, culture positive by any method: n = 778 (31.8%), culture negative by all methods: n = 1603 (65.5%), contaminated samples without a positive parallel culture: n = 65 (2.7%).

2446 sputum samples were processed (figure 1). A total of 778 samples (31.8%) yielded a positive TB culture by at least one of the three culture methods (LJ, conventional MODS, and MODS Kit), of which 674 samples (27.6% of all samples, 86.6% of positive samples) were positive by all three methods. Ten (0.4% of all samples, 1.3% of all positive samples) were positive in MODS Kit only, 1 (0.04% of all samples, 0.1% of all positive samples) was positive in conventional MODS only, and 56 (2.3% of all samples, 7.2% of all positive samples) were positive by LJ culture only. A further 21 (2.7%) additional samples were positive by both MODS Kit method and LJ culture (with scanty growth, <20 colonies, in 50%), but not by the conventional MODS assay. 1603 (65.5%) were culture negative by all three methods, and 65 samples (2.7%) were contaminated by at least one method, without a parallel positive culture.

Of the 56 samples that were positive by LJ culture only, 50 (89.3%) were reported as demonstrating only “scanty” (<20 colonies) growth. Of the 87 samples that were positive by LJ and/or MODS Kit, but negative by conventional MODS, one third of samples (n = 29) were classified as “indeterminate” in the parallel conventional MODS assay on first processing (meaning growth was observed but not sufficient to reach the threshold of 2 or more CFU in both drug free wells) and all but one were negative after reprocessing (including a second decontamination step) and repeat culture performed according to the protocol. Similarly, 50% (5 of 10) of the samples that were positive only by the MODS Kit, but not by either LJ culture or conventional MODS, had an indeterminate result when first processed by conventional MODS.

### Performance of MODS Kit for detection of *M. tuberculosis* in sputum

**Figure 2:**
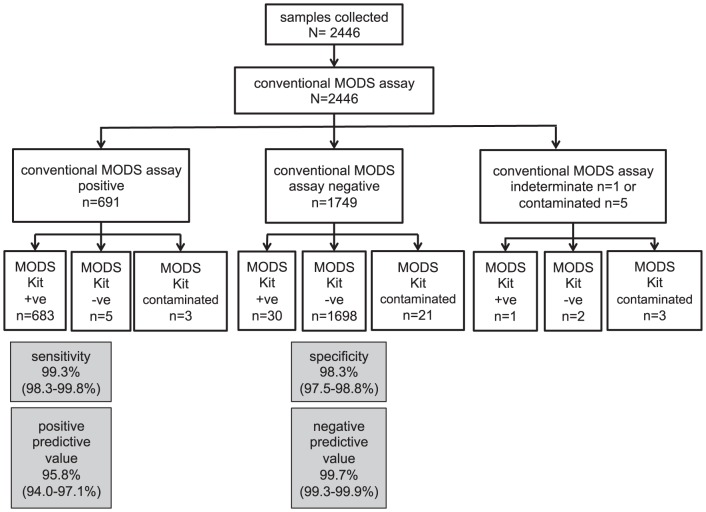
STARD (STAndards for the Reporting of Diagnostic accuracy) flow diagram for MODS Kit for TB detection. Kappa value 0.94, agreement 97.4%. +ve = TB culture positive, -ve = TB culture negative. Contaminated & indeterminate results are culture outcomes following reprocessing if necessary according to protocol.

Using the conventional MODS assay as the reference standard, the sensitivity, specificity, positive, and negative predictive values (and associated 95% confidence intervals) achieved with the MODS Kit were 99.3% (98.3–99.8%), 98.3% (97.5–98.8%), 95.8% (94.0–97.1%), and 99.7% (99.3–99.9%), with a kappa value of 0.94 (figure 2). Median (IQR) time to culture-positivity was 10 (9–13) days for conventional MODS and 8.5 (7–11) for MODS Kit (p<0.01).

### Performance of MODS Kit in determining direct isoniazid and rifampicin susceptibility compared to conventional MODS

Of the 683 samples yielding positive cultures by both MODS methods, four samples (0.6%) were excluded from the DST analysis because the drug containing wells could not be read (three due to culture contamination and one due to indeterminate *M. tuberculosis* growth). For 665 (97.9%) of the remaining 679 samples included in the analysis susceptibility test results for isoniazid and rifampicin were concordant (Table 1).

**Table 1:** Direct DST result by MODS Kit and conventional MODS.

		Conventional MODS assay				total
		Drug susceptible (RIF & INH)	INH monoresistant	RIF monoresistant	MDRTB	
MODS Kit DST	Drug susceptible (RIF & INH)	531	4	0	0	535
	INH monoresistant	1	44	0	1	46
	RIF monoresistant	4	1	17	0	22
	MDRTB	0	1	2	73	76
total		536	50	19	74	679

concordant DST, n = 665 (shaded grey).

discordant DST, n = 14 (no shading).

#### Rifampicin – MODS kit vs. conventional MODS

For rifampicin DST, 580 of 586 samples determined as rifampicin susceptible by conventional MODS assay were also identified as susceptible by MODS Kit, and 92 of 93 samples determined as rifampicin resistant by the conventional method were also identified as rifampicin resistant by MODS Kit (regardless of isoniazid DST result, Table S1a). The corresponding sensitivity and specificity for detection of rifampicin resistance (with 95% confidence intervals) were 98.9% (94.2–100.0%) and 99.0% (97.8–99.6%), respectively. Positive and negative predictive values were 93.9% (87.1–97.7%) and 99.8% (99.0–100.0%), respectively, for determination of rifampicin resistance, with a kappa value of 0.96.

#### Isoniazid – MODS kit vs. conventional MODS

For isoniazid DST 552 of 555 samples determined as isoniazid susceptible by the conventional MODS assay were also identified as susceptible by MODS Kit, and 119 of 124 samples determined as isoniazid resistant by conventional MODS were also identified as isoniazid resistant by MODS Kit (regardless of rifampicin DST results, Table S1b). The corresponding sensitivity and specificity for detection of isoniazid resistance (with 95% confidence intervals) were 96.0% (90.8–98.7%) and 99.5% (98.4–99.9%), respectively. Positive and negative predictive values were 97.5% (93.0–99.5%) and 99.1% (97.9–99.7%), respectively, for determination of isoniazid resistance, with a kappa value of 0.96 (data in Table 1).

### Concordance of MODS Kit with indirect proportion method testing in determining direct isoniazid and rifampicin susceptibility

704 samples were culture positive by both the MODS Kit and another culture method. Within these samples the drug susceptibility profile for rifampicin and isoniazid could be compared between the MODS Kit and the indirect proportions method (Table 2 and Figure S1), with the exception of one culture with indeterminate *M. tuberculosis* growth in the MODS Kit drug-containing wells. Ten additional samples were culture positive only by the MODS Kit from which it was possible to subculture seven strains for comparative testing by the indirect proportions method. Eight samples subject to discrepant analysis were subsequently found by Genotype MTB-DR plus to be either non-tuberculous mycobacteria (n = 3) or contain mixed *M. tuberculosis* strains (ie a mixed DST result, 4 samples containing strains both sensitive and resistant to rifampicin and one containing strains both sensitive and resistant to isoniazid) and were thus excluded leaving 702 paired MODS Kit – indirect DST results for comparison.

**Table 2:** Direct DST result by MODS Kit compared with indirect DST result by proportions method (with discrepant analysis by Genotype MTB-DR plus).

		Reference standard indirect DST				total
		Drug sensitive (RIF & INH)	INH monoresistant	RIF monoresistant	MDRTB	
MODSKit DST	Drug sensitive (RIF & INH)	549	3	0	1	553
	INH monoresistant	2	41	0	1	44
	RIF monoresistant	1	2	22	1	26
	MDRTB	2	1	1	75	79
total		554	47	23	78	702

Data in table indicate consolidated reference indirect test result after discrepant analysis employing Genotype MTB-DR plus line probe assay as arbiter test (Genotype MTB-DR plus determined final true result in those samples for which MODS Kit and proportions method were discordant).

concordant DST, n = 687 (shaded grey).

discordant DST, n = 15 (no shading).

The rifampicin/isoniazid assignment as susceptible/susceptible, susceptible/resistant, resistant/susceptible, or resistant/resistant was concordant between MODS Kit direct DST and the (indirect) proportion method for 97.9% (n = 687) of the 702 comparable results.

#### Rifampicin – MODS kit vs. indirect reference DST (consolidated standard result)

The sensitivity and specificity for detection of rifampicin resistance, (with 95% confidence intervals) in comparison to the reference standard were 98.0% (93.0–99.8%) and 99.0% (97.8–99.6%), respectively. Positive and negative predictive values were 94.3% (88.0–97.9%) and 99.7% (98.8–100.0%), respectively, for determination of rifampicin resistance. MODS Kit-proportions method concordance for rifampicin resistance was 98.9% (kappa 0.95, Table S2a).

#### Isoniazid – MODS kit vs. indirect reference DST (consolidated standard result)

The sensitivity, and specificity for detection of isoniazid resistance (with 95% confidence intervals) were 94.4% (88.8–97.7%) and 99.1% (98.0–99.7%), respectively. Positive and negative predictive values were 95.9% (90.8–98.7%) and 98.8% (97.5–99.5%), respectively, for determination of isoniazid resistance. MODS Kit-proportions method concordance for isoniazid resistance was 98.3% (kappa 0.94, Table S2b).

### Culture contamination and indeterminate results

The proportion of initially contaminated samples was 6.0% and 3.0% in LJ and the MODS Kit cultures, respectively (p<0.01); this MODS Kit rate was not significantly different (p = 0.07) to the 2.2% of conventional MODS cultures that were contaminated (146, 73, and 53 of total 2446 samples, respectively). After reprocessing, the proportion of contaminated cultures dropped to 2.3% for LJ, 1.1% for MODS Kit, and 0.2% (p<0.01) for conventional MODS culture (all significantly different p<0.01, N = 56, 27 and 5 of total 2446 samples, respectively).

After initial processing 0.5% (N = 12) of all MODS Kit cultures and 1.6% (N = 40) of all conventional MODS cultures were reported as indeterminate (p<0.01); however, after reprocessing in accordance with the protocol only 1 result remained indeterminate (in conventional MODS).

## Discussion

This study provides the first reported data comparing the MODS Kit with conventional, non-commercial MODS and other TB reference diagnostic assays. This large evaluation study confirms that the performance of the MODS Kit under field conditions is virtually indistinguishable from the existing MODS assay. When compared with conventional, non-commercial MODS the MODS Kit delivered high sensitivity and specificity for TB detection at 99.3% and 98.3%, respectively, for detection of rifampicin resistance at 98.9% and 99.0%, respectively, and for detection of isoniazid resistance at 96.0% and 99.5%, respectively. Kappa values for agreement between conventional MODS and the MODS Kit were high at 0.94 for TB detection and 0.96 for detection of both rifampicin and isoniazid resistance. An important characteristic of diagnostic tests is that only a small proportion of tests deliver indeterminate results–here, a high proportion of tests (98.9%, 2419 of 2446) gave a definitive positive or negative result by MODS Kit after all samples were processed according to the protocol.

Adding to the large body of evidence validating the use of direct rifampicin and isoniazid DST in the MODS assay [14], the excellent performance of the MODS Kit for rifampicin and isoniazid direct DST was confirmed by comparison to indirect DST by the proportions method with 97.9% samples giving a concordant result between the two methods for combined isoniazid and rifampicin susceptibility. Of the 22 samples for which MODS kit and the proportions method were not concordant, discrepant analysis by an additional molecular test (Genotype MTB-DR plus) was performed. The results from this third arbitrator test were evenly split, suggesting that neither MODS kit nor the proportions method out-performed the other.

In this study 7.2% (56 of 778) of samples positive by any method were positive only by LJ culture, a finding that is unusual and rarely reported in studies comparing MODS liquid culture with LJ solid media culture. The finding that the majority (49 of 56, 87.5%) had only scanty growth recorded in this subgroup might raise concern that this represented cross-contamination (i.e. false-positive LJ culture), but this cannot be proven. Alternatively, it is conceivable that MODS failed to detect small quantities of bacilli present in paucibacilliary samples that LJ was able to detect, though the reverse would usually be expected. Unequal division of sputum samples for clinical diagnostic studies is a recognised potential source of bias, but there is no reason to believe that any systematic error in sample division took place. Therefore, one would expect as many MODS positive/LJ negative samples as vice versa if this was the cause. One potential explanation could be the inadvertent inclusion of samples from patients who were on treatment, for which LJ culture may sometimes offer a slight advantage [15]. At the time of this study MODS was offered as a diagnostic test as part of the National TB Programme in Peru only for pre-treatment samples (on-treatment samples have since been included in the programmatic guideline), but it is possible that clinicians submitted on-treatment samples for “diagnostic” testing to obtain a more rapid result. Though conduct of this study on anonymised samples in the operational setting of a routine laboratory significantly strengthens the external validity of the study findings, a drawback is the lack of accompanying patient metadata to help tease out these observations. Laboratory factors that may reduce TB detection rates in MODS include over-enthusiastic sample decontamination or inadequate broth supplementation. Initial contamination rates after first processing were 2.2%, 3.0%, and 6.0% in conventional MODS, the MODS Kit, and LJ culture, respectively, which, for MODS culture falls in the recommended range of 2–3% of samples [12]. Final contamination rates (after all contaminated samples were decontaminated for a second time according to standard protocol) observed in both the conventional MODS and the MODS Kit assay, were 0.20% and 1.10%, respectively (p<0.01), lower than the recommended contamination rate, and this may have adversely affected the yield of positive mycobacterial culture results [12].

The MODS Kit has been designed to improve the capability of busy TB laboratories often located in resource-constrained settings to provide rapid, reliable and cost-effective diagnostic testing whilst minimising workload and protecting the safety of laboratory staff. This study was performed in a government laboratory and is therefore likely to accurately reflect the performance of the MODS Kit in a working laboratory. Clinical and molecular studies to exclude cross contamination were not part of this study; however, a previous detailed study that compared LJ and MODS culture cross contamination rates found low rates and no significant differences between the 2 methods [16]. The novel, self-sealing transparent silicone plate lid in the MODS kit prevents exit, entry, or between well spill over of any culture contents and on closure provides absolute security against cross-contamination after samples are inoculated into wells of the 24 well plate.

In a large operational study in Peru, conventional MODS outperformed automated mycobacterial culture and culture on LJ medium with sensitivity and specificity of the MODS assay for TB detection (in a median of 7 days) of 97.3% and 99.7%, respectively [4]. Compared to the current study contamination rates were higher at 8.1% and 14.2% for initial samples cultured by MODS and LJ, respectively; after samples were decontaminated, and re-cultured for a second time if needed, the rates were similar to this study at 0.2% and 1.5%, respectively [4]. Other studies have also reported the reliable and rapid performance of the MODS assay compared to other existing tests [2], [3], [17]–[19]. Low contamination rates in this study may indicate that the decontamination procedure is killing too many tuberculous bacilli along with the contaminating organisms. Centrifugation and decontamination have been demonstrated to kill approximately 78% of organisms where samples have been cultured in liquid media and colonies counted [20].

Other possibilities for reduced growth in the MODS assay include inadequate enrichment of the broth, although there is nothing to suggest that this is the case from review of the reagents used by the laboratory and all internal positive and negative quality controls gave appropriate expected results. Median times to positivity for both MODS methods in this study were short at 8.5 days and 10 days, but were marginally longer than as previously reported (7 days) in a research laboratory setting [4]. This may be explained by inevitable time constraints for plate reading in this busy service laboratory and a tendency to defer calling a culture positive until MTB growth is unequivocal.

MODS was originally developed as a low-cost, non-commercial assay with a view to delivering open access “laboratory freeware” to all, which was an attribute thought to be particularly useful for high TB burden countries with limited resources and huge unmet diagnostic needs. The non-commercial, non-kit aspect proved to be less of a facilitating factor and rather more of an obstacle to uptake, dampening enthusiasm in some circles. The process of MODS endorsement by the World Health Organisation started with an expert group meeting in April 2009 followed by a Strategic and Technical Advisory Group on TB (STAG-TB) recommendation in November 2009 that finally resulted in the issuing of a WHO policy recommendation in 2011, which was seen by almost nobody given the lack of an associated press release and the publishing of the recommendation to an unlinked, thus hidden, url.

The contrast with the speed of passage of commercial diagnostics with substantially less published data is stark. Non-commercial diagnostics have an unwarranted image problem and users appreciate the convenience that a single supplier provides, particularly if some of the preparatory work (in this case media and drug preparation) is already done. The key question for MODS then becomes, “can a commercial version of MODS deliver an equally cost-effective solution?” This study unequivocally affirms the effectiveness, but what of the cost? A cost comparison study in Peru concluded that the conventional MODS assay cost $5 USD per patient [21]. The pre-shipping list price of the CE-marked MODS Kit, produced under Good Manufacturing Process (GMP) conditions, has been put at under $6 USD per patient, which is both highly competitive with conventional MODS and unprecedented for commercial delivery of liquid culture with both rifampicin and isoniazid DST.

This study clearly demonstrates that the MODS Kit performs at least as well as the existing non-commercial MODS assay for the rapid and reliable detection of TB and diagnosis of MDRTB direct from sputum. The kit provides all the required quality-controlled reagents in a single purchase order, enhances biosafety for laboratory technicians, and is well suited to resource poor settings where TB is common. Laboratory staff reported that the kit was easy to use for both setting up the culture and reading of the plates. The scale-up of implementation of rapid molecular tests highlights the importance of maintaining conventional culture facilities, which are increasingly required for detection of treatment failure and drug susceptibility testing beyond rifampicin alone. The MODS platform in both its conventional non-commercial form and now as the MODS Kit provides a reliable and cost effective way of delivering this. An extended MODS assay for second line drug susceptibility testing and XDR-TB diagnosis is an exciting near-future possibility [22], [23]. Incorporation of appropriate concentrations of additional TB drugs into the MODS Kit format would be simple, offering a straightforward follow-on phenotypic DST for second line agents for patients found to be harbouring organisms resistant to isoniazid or rifampicin (including those identified by molecular assays including Xpert MTB/RIF). The results of this evaluation and the improvement on procurement and biosafety issues provide an important opportunity to use wide scale introduction of the MODS kit as a vehicle for greatly expanding equitable access to first and second line DST in settings where the need is greatest.

## Supporting Information

Figure S1 **Paired samples available for comparison of MODS Kit and indirect proportions methods DST results.** Total samples: N = 2446, culture positive by any method: n = 778 (31.8%), culture negative by all methods: n = 1603 (65.5%), contaminated samples without a positive parallel culture: n = 65 (2.7%).
(EPS)

Table S1 Table S1a: Concordance of MODS Kit with conventional MODS method testing in determining direct isoniazid susceptibility, regardless of rifampicin DST result. Table S1b: Concordance of MODS Kit with conventional MODS method testing in determining direct rifampicin susceptibility, regardless of isoniazid DST result.
(PDF)

Table S2 Table S2a: Concordance of MODS Kit with indirect DST result by proportions method (with discrepant analysis by Genotype MTB-DR plus) in determining direct isoniazid susceptibility, regardless of rifampicin DST result. Data in table indicate consolidated reference indirect test result after discrepant analysis employing Genotype MTB-DR plus line probe assay as arbiter test (Genotype MTB-DR plus determined final true result in those samples for which MODS Kit and proportions method were discordant). Table S2b: Concordance of MODS Kit with indirect DST result by proportions method (with discrepant analysis by Genotype MTB-DR plus) in determining direct rifampicin susceptibility, regardless of isoniazid DST result. Data in table indicate consolidated reference indirect test result after discrepant analysis employing Genotype MTB-DR plus line probe assay as arbiter test (Genotype MTB-DR plus determined final true result in those samples for which MODS Kit and proportions method were discordant).
(PDF)

File S1 **Original database for MODS Kit study.**
(CSV)

File S2 **Key to variable labels and codes used for MODS Kit study.**
(PDF)
