# Supplementary material for: A Field Evaluation of the Hardy TB MODS Kit™ for the Rapid Phenotypic Diagnosis of Tuberculosis and Multi-Drug Resistant Tuberculosis
Source: PLoS One. 2014 Sep 16;9(9):e107258. doi: 10.1371/journal.pone.0107258 (PMC4167337; doi:10.1371/journal.pone.0107258)
Supplement: Table S1 — a: Concordance of MODS Kit with conventional MODS method testing in determining direct isoniazid susceptibility, regardless of rifampicin DST result. Table S1b: Concordance of MODS Kit with conventional MODS method testing in determining direct rifampicin susceptibility, regardless of isoniazid DST result. (PDF) [file pone.0107258.s002.pdf]

Table S1a

			conventional MODS DST		
			isoniazid		total
			susceptible	resistant	
Mods Kit DST	isoniazid	susceptible	552	5	557
		resistant	3	119	122
	total		555	124	679

Table S1b

			conventional MODS DST		
			rifampicin		total
			susceptible	resistant	
Mods Kit DST	rifampicin	susceptible	580	1	581
		resistant	6	92	98
	total		586	93	679
