# Supplementary material for: A Field Evaluation of the Hardy TB MODS Kit™ for the Rapid Phenotypic Diagnosis of Tuberculosis and Multi-Drug Resistant Tuberculosis
Source: PLoS One. 2014 Sep 16;9(9):e107258. doi: 10.1371/journal.pone.0107258 (PMC4167337; doi:10.1371/journal.pone.0107258)
Supplement: Table S2 — a: Concordance of MODS Kit with indirect DST result by proportions method (with discrepant analysis by Genotype MTB-DR plus) in determining direct isoniazid susceptibility, regardless of rifampicin DST result. Data in table indicate consolidated reference indirect test result after discrepant analysis employing Genotype MTB-DR plus line probe assay as arbiter test (Genotype MTB-DR plus determined final true result in those samples for which MODS Kit and proportions method were discordant). Table S2b: Concordance of MODS Kit with indirect DST result by proportions method (with discrepant analysis by Genotype MTB-DR plus) in determining direct rifampicin susceptibility, regardless of isoniazid DST result. Data in table indicate consolidated reference indirect test result after discrepant analysis employing Genotype MTB-DR plus line probe assay as arbiter test (Genotype MTB-DR plus determined final true result in those samples for which MODS Kit and proportions method were discordant). (PDF) [file pone.0107258.s003.pdf]

Table S2a

			reference DST		
			isoniazid		total
			susceptible	resistant	
Mods Kit DST	isoniazid	susceptible	572	7	579
		resistant	5	118	123
	total		577	125	702

Table S2b

			reference DST		
			rifampicin		total
			susceptible	resistant	
Mods Kit DST	rifampicin	susceptible	595	2	597
		resistant	6	99	105
	total		601	101	702
