# Supplementary material for: A Field Evaluation of the Hardy TB MODS Kit™ for the Rapid Phenotypic Diagnosis of Tuberculosis and Multi-Drug Resistant Tuberculosis
Source: PLoS One. 2014 Sep 16;9(9):e107258. doi: 10.1371/journal.pone.0107258 (PMC4167337; doi:10.1371/journal.pone.0107258)
Supplement: File S2 — Key to variable labels and codes used for MODS Kit study. (PDF) [file pone.0107258.s005.pdf]

Database key

kmods=MODS Kit cMODS=conventional MODS lj=Lowestein Jensin Culture, pm=proportions method on agar, hain=Genotype MTBDRplus test, r “reference DST” is described in methods

Variable	Description	codes for data
study_code	random code assigned to samples	
smear_study	sputum auramine microscopy result	0=negative, 1=+, 2=++,3=+++,666=paucibacillary
kmods_process_date	culture start date MODS Kit	
kmods_result_date	culture result date MODS Kit	
kmods_repro_yn	culture reprocessed MODS Kit	0=not reprocessed 1=reprocessed (if contaminated/indeterminate)
kmods_repro_date	reprocessed culture start date MODS Kit	
kmods_repro_result_date	reprocessed culture result date MODS Kit	
kmods_result_final	final MODS Kit result	
kmods_result_date_final	date of final MODS Kit result	0=negative, 1=positive, 66=contaminated
kmods_rif_final	rifampicin result MODS Kit	0=rifampicin susceptible, 1=rifampicin resitant
kmods_inh_final	isoniazid result MODS Kit	0=isoniazid susceptible, 1=isoniazid resitant
cmods_lj_process_date	culture start date conventional MODS assay & LJ culture	
cmods_result_date	culture result date conventional MODS assay	
cmods_repro_yn	culture reprocessed conventional MODS assay (contaminated or indeterminate)	0=not reprocessed 1=reprocessed (if contaminated/indeterminate)
cmods_repro_date	reprocessed culture start date conventional MODS assay	
cmods_repro_result_date	reprocessed culture result date conventional MODS assay	
cmods_result_final	final conventional MODS result	

cmods_result_date_final	date of final conventional MODS assay result	0=negative, 1=positive, 66=contaminated
cmods_rif_final	rifampicin result conventional MODS	0=rifampicin susceptible, 1=rifampicin resitant
cmods_inh_final	isoniazid result conventional MODS	0=isoniazid susceptible, 1=isoniazid resitant
lj_result_date	culture result date LJ culture	
lj_repro_date	culture reprocessed LJ culture	0=not reprocessed 1=reprocessed (if contaminated/indeterminate)
lj_repro_result_date	reprocessed culture start date LJ culture	
lj_result_final01	final LJ culture result	0=negative, 1=positive, 66=contaminated
lj_result_date_final	date of final LJ culture result	
pm_process_date	proportions method start date	
pm_inh_result	proportions method inh DST result	0=isoniazid susceptible, 1=isoniazid resitant
pm_rif_result	proportions method rif DST result	0=rifampicin susceptible, 1=rifampicin resitant
pm_result_date	proportions method result date	
pm_dst_result	proportions method DST result	SENS=rif & inh susceptible, MDR=rif & inh resistant, INH-R=inh monoresistant, RIF-R-rif monoresistant
hain_process_date	Hain test start date	
hain_inh_result	Hain test inh DST result	0=isoniazid susceptible, 1=isoniazid resitant
hain_rif_result	Hain test rif DST result	0=rifampicin susceptible, 1=rifampicin resitant
hain_result_date	Hain test result date	
hain_dst_result	Hain test DST result (and comments)	SENS=rif & inh susceptible, MDR=rif & inh resistant, INH-R=inh monoresistant, RIF-R-rif monoresistant
dst_reference	reference standard DST result	SENS=rif & inh susceptible, MDR=rif & inh resistant, INH-R=inh monoresistant, RIF-R-rif monoresistant
rif_res_reference	reference standard inh DST result	0=isoniazid susceptible, 1=isoniazid resitant
inh_res_reference	reference standard DST result	0=rifampicin susceptible, 1=rifampicin resitant
